# Supplementary material for: Proteome-wide determinants of co-translational chaperone binding in bacteria
Source: Nat Commun. 2025 May 10;16:4361. doi: 10.1038/s41467-025-59067-9 (PMC12065913; doi:10.1038/s41467-025-59067-9)
Supplement: Supplementary file 1 — Supplementary Information [file 41467_2025_59067_MOESM1_ESM.pdf]

Supplementary information

Supplementary figures:

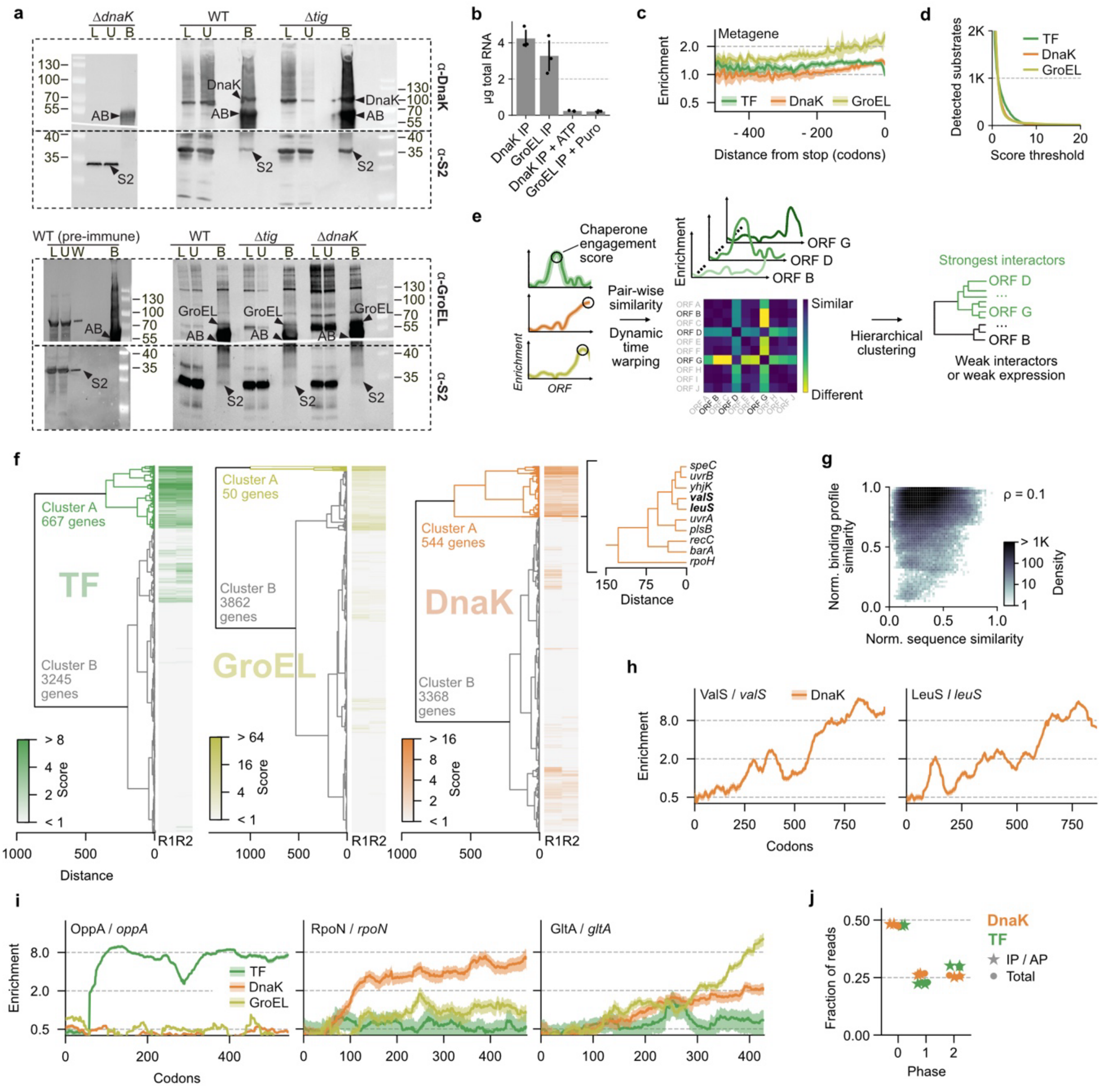

Supplementary Fig. 1 | Quantification of chaperone interactions. a, Western blot analysis of DnaK-ribosome nascent chain complexes (RNC) and GroEL-RNC immunoprecipitations (IP) using antisera specific for DnaK (α-DnaK) or GroEL (α-GroEL) and the ribosomal protein S2 (α-S2). L: lysate, W: wash, U: unbound, B: bound fraction. AB: antibody used in the DnaK / GroEL IP. **b,** Amount of co-purified ribosomes upon DnaK or GroEL IP, determined by RNA quantification (Bioanalyzer). The control shows co-purified ribosomes obtained after ATP (DnaK IP) or puromycin treatment (GroEL IP). Mean (bar height) and standard deviation (SD, error bars) are shown for three replicates (dots). **c**, Genome-wide (n = 3961) metagene enrichment profiles for TF (green), DnaK (orange) and GroEL (olive). Profiles are aligned to the stop codon. Solid lines and shadows indicate means and 95 % CI, respectively. **d**, Threshold plot comparing the number of substrates versus an increasing engagement score threshold for TF, DnaK and GroEL. **e**, Continuation of Fig. 1a. Schematic of the analysis of SeRP data. The highest values of the low CI border

(black circle) are used to determine chaperone engagement scores (left). Then, low CI borders of all genes were compared pair-wise using dynamic time warping, a method developed to find similarities between time-series (explicitly spoken language) with different lengths (mid). The resulting 2D similarity matrices were then linearized and sorted by hierarchical clustering using the Ward variance minimization algorithm[1] (right). **f**, Cluster analysis of TF (green), GroEL (olive) and DnaK (orange) bound translatomes. Heatmaps show both replicates of engagement scores based on Supplementary Data 1. Inset plot on the right shows the strongest enriched and well separated subcluster of DnaK substrates. **g**, Scatterplot for all gene-gene combinations in clusters A for TF, DnaK and GroEL (n = 365921 pairs). Sequence similarities between two genes were obtained by the PairwiseAligner Module from Biopython[2] and normalized by the length of the target sequence to obtain values between 0 and 1. Chaperone enrichment similarities were calculated by dynamic time warping and normalized by setting the most similar pair to one and the least similar pair to zero. ρ indicates the Spearman correlation coefficient. **h**, DnaK enrichment profiles for ValS (left) and LeuS. (right). Solid lines indicate averages, shadows indicate the 95 %. **i**, Gene-specific chaperone enrichment profiles of TF (OppA), DnaK (RpoN) and GroEL (GltA). Solid lines indicate averages, shadows indicate the 95 %. **j**, Read periodicity of individual total translatomes and chaperon specific datasets. Source data are provided as a Source Data file.

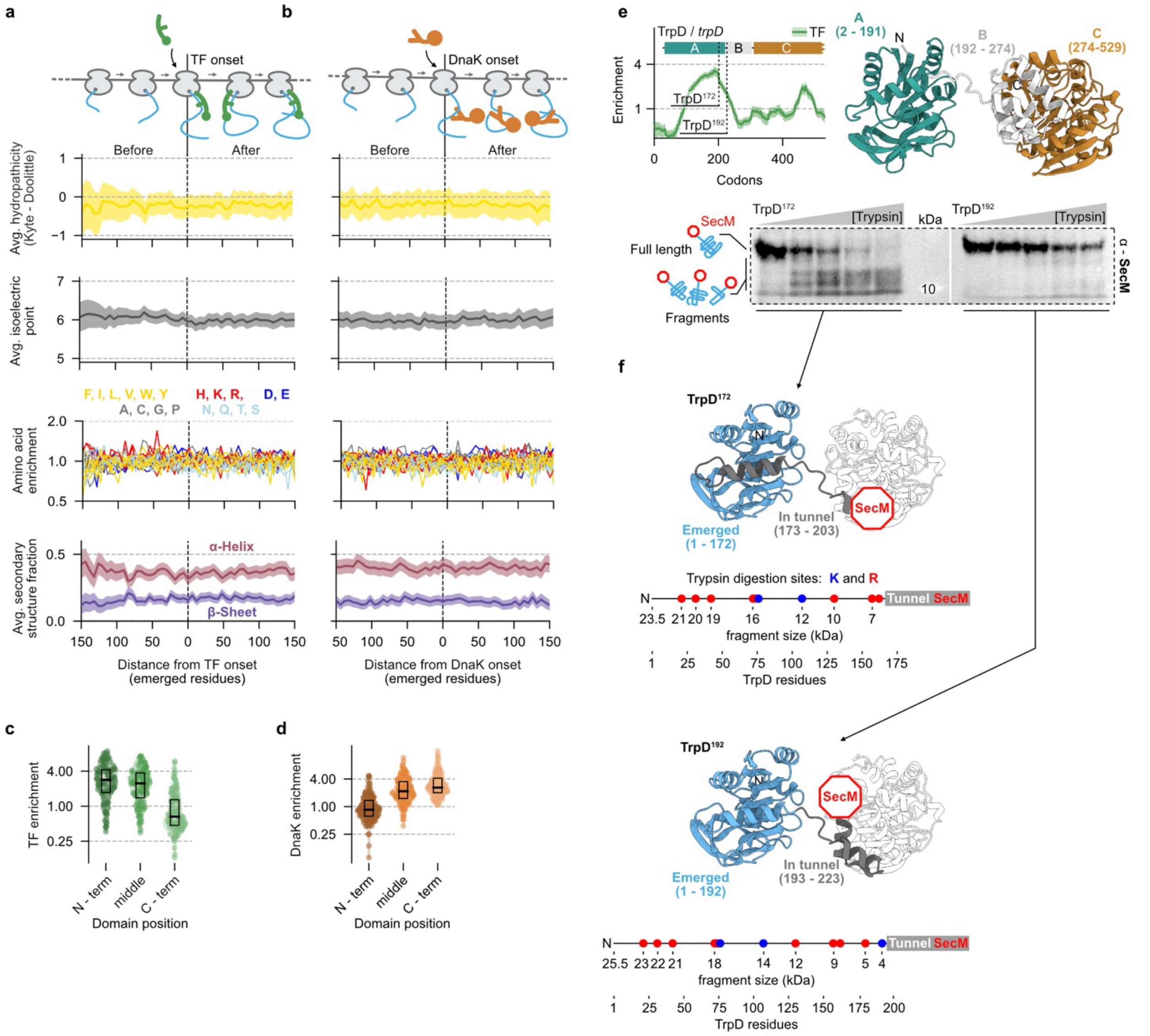

Supplementary Fig. 2 | Features of exposed nascent chain segments upon TF and DnaK engagement. a and b, Averaged nascent chain features before and after chaperone engagement. Positions are shifted by 30 amino acids to the 5' end of transcripts to account for the ribosomal exit tunnel. TF (a, n = 436) and DnaK (b, n = 353) cytosolic, high affinity substrates are shown. The position of initial chaperone engagement is indicated by a dashed line. Regions of 6 consecutive residues were binned and averaged among all included genes (solid lines). Shadows indicate 95 % CI. From top to bottom: hydropathicity (Kyte-Doolittle scale[3]), isoelectric point, enrichment of an amino acid compared to its frequency among all strong chaperone substrates, α-helix and β-sheet content taken from AlphaFold predictions. **c**, Maximal TF enrichment within N-terminal, middle, and C-terminal domains of 114 cytosolic high affinity TF substrates comprising at least three domains. Domains shorter than 100 amino acids were excluded. Black bars indicate medians and boxes indicate the interquartile range. **d**, same as c but for 152 cytosolic high affinity DnaK substrates. **e**, TF engagement profile to nascent TrpD (top left). Colored bars above show the tunnel normalized CATH domain annotation. Solid line indicates the average, shadow indicates the 95 % CI. AlphaFold prediction of the TrpD structure (AF-P00904, top right) with colored domain annotation,

numbers show amino acid positions. Folding states of nascent chains were probed by limited proteolysis using increasing concentrations of trypsin (0, 77, 193, 483 and 1208 ng/ml f.c.) followed by western blot analysis using α-SecM primary antibodies. RNCs exposing N-terminal 172 amino acids of TrpD (bottom left). RNCs exposing N-terminal 192 amino acids of TrpD (bottom right). **f**, AlphaFold prediction of the TrpD structure. Tunnel exposed residues in both TrpD fragments are colored in blue, Residues inside the ribosomal exit tunnel in the TrpD-SecM fragments are colored in grey, the SecM stalling tag is shown in red; numbers show amino acid positions. Lysine (K, blue) and arginine (R, red) trypsin cleavage sites within TrpD with corresponding C-terminal tryptic fragment sizes (kilodalton, kDa) are shown below every structure. Source data are provided as a Source Data file.

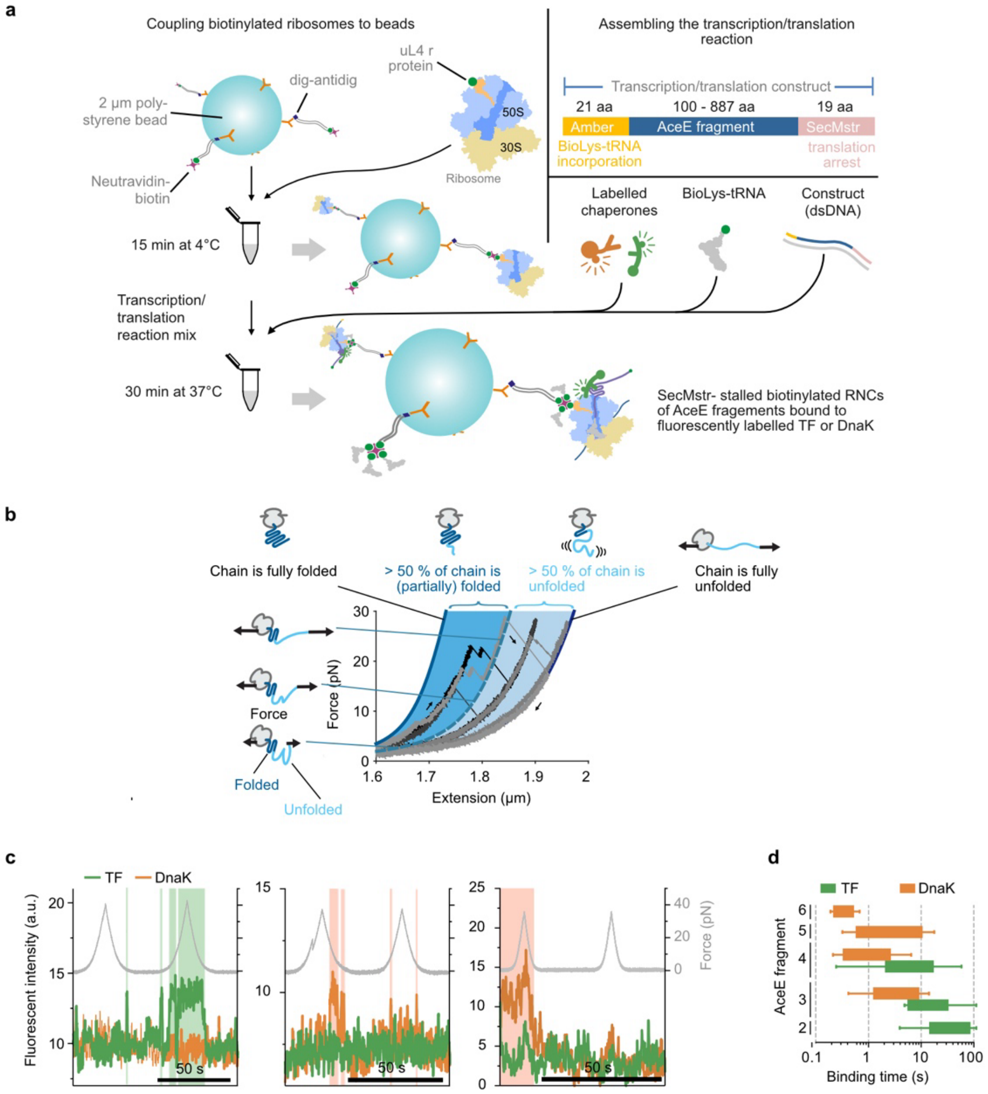

Supplementary Fig. 3 | Characterization of nascent chain folding states using optical tweezers coupled with fluorescence microscopy. a, Optical tweezer schematic experimental assembly. First biotinylated ribosomes were coupled to Neutravidin-DNA coated beads, then SecMstr-stalled biotinylated nascent chains of AceE fragments were synthesized in the presence of TF and/or DnaK using a customized PURE expression system. **b**, Example force-extension data revealing the folded state of the nascent chain of AceE fragment 5. Two classes of folding states are depicted, one in which the majority of the nascent chain is folded and the other where the majority of it is unfolded. The accompanying cartoon illustrates the varying degrees of compaction of the nascent chain during the pulling and relaxation cycles. **c**, Simultaneous force-fluorescence measurements of individual AceE nascent chain fragments interacting with TF (green shadow) and DnaK (orange shadow). **d**, Binding times of TF (green) and DnaK (orange) depending on the AceE fragment length. n: TF AceE fragment 2-6: 27, 15, 18, 0, 0. DnaK AceE fragment 2-6: 0, 6, 10, 21, 4. Boxes indicate the interquartile range, whiskers are drawn down to the 10th and 90th percentile. Source data are provided as a Source Data file.

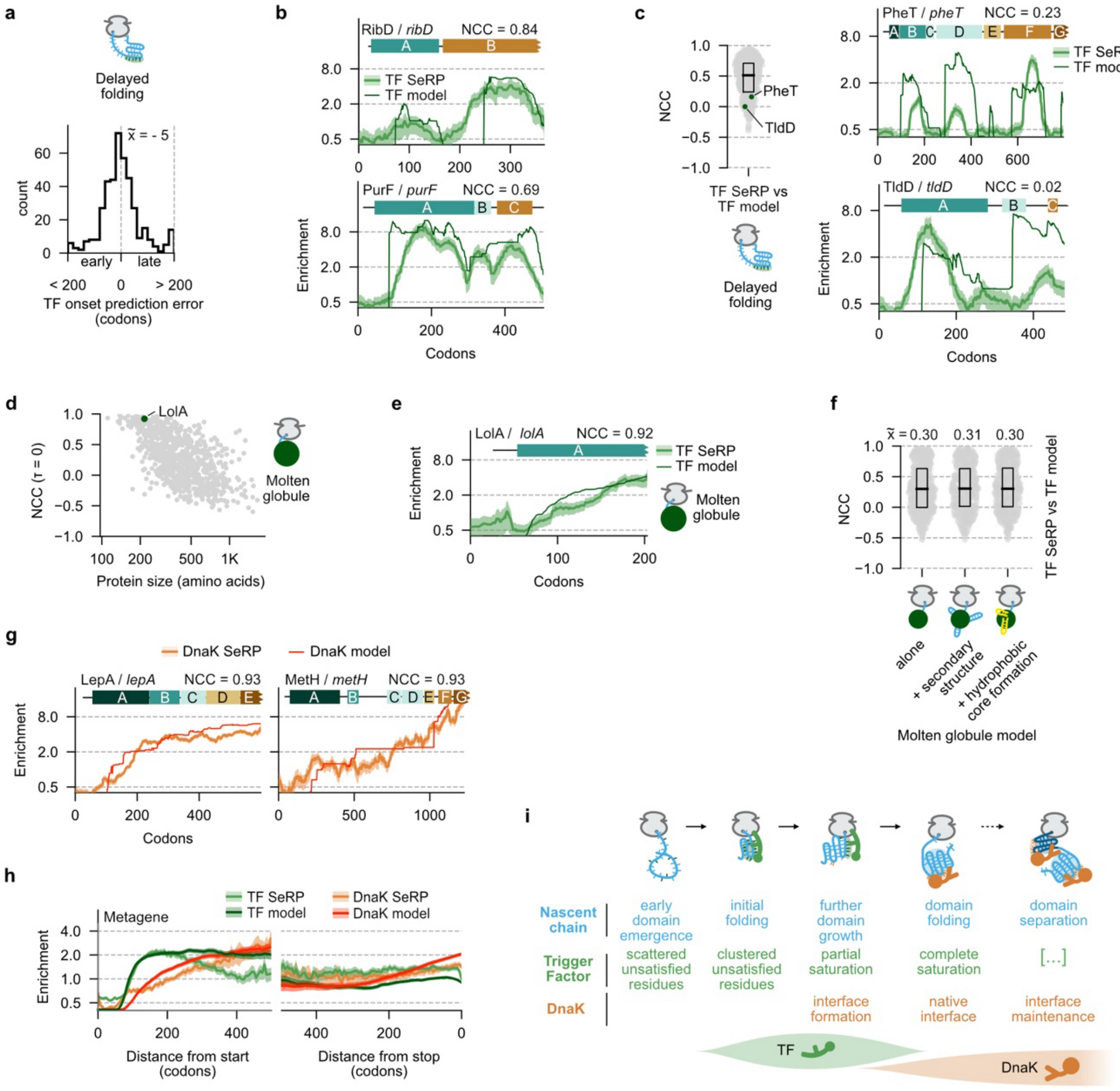

Supplementary Fig. 4 | Computational prediction of TF and DnaK binding to nascent chains. a. Computationally predicted TF engagement onsets are compared against TF engagement onsets derived from SeRP (n = 429, median is shown at the top). **b**, TF delayed folding model predictions (dark green) and SeRP based TF enrichment profile (green) for RibD (top) and PurF (bottom). Solid thick lines indicate averages, shadows indicate the 95 % CI. **c**, Repetition of Fig. 4b, with NCCs of PheT and TldD highlighted in green (left). TF delayed folding model predictions (dark green) and SeRP based TF enrichment profile (green) for PheT (top right) and TldD (bottom right). Solid thick lines indicate averages, shadows indicate the 95 % CI. **d**, Protein size (x-axis) plotted against the normalized cross correlation at tau = 0 (NCC, y-axis) for TF binding prediction based on the molten globule nascent chain folding model. LolA is highlighted in dark green. **e**, TF molten globule model predictions (dark green) and SeRP based TF enrichment profile (green) for LolA. Solid thick line indicates the average, shadow indicates the 95 % CI. **f**, NCC comparing "molten globule" TF model predictions with the SeRP derived low CI boundary from strong cytosolic TF substrates (n = 429 for all columns). Black bars and top numbers (x̃) indicate the median NCC while boxes indicate interquartile ranges. **g**, DnaK model predictions (dark red) and SeRP based DnaK enrichment

profile (orange) for LepA (left) and MetH (right). Solid thick lines indicate averages, shadows indicate the 95 % CI. **h**, Metagene analysis of the TF model (dark green) and the DnaK model (dark red) for 2081 cytosolic genes. As reference, SeRP derived metagene enrichment profiles for TF (green) and DnaK (orange) are shown. Gene alignment to the start and stop codon is shown left and right, respectively. Solid lines and shadows indicate means and 95 % CI, respectively. **i**, Scheme of co-translational folding support for cytosolic proteins by TF and DnaK. Source data are provided as a Source Data file.

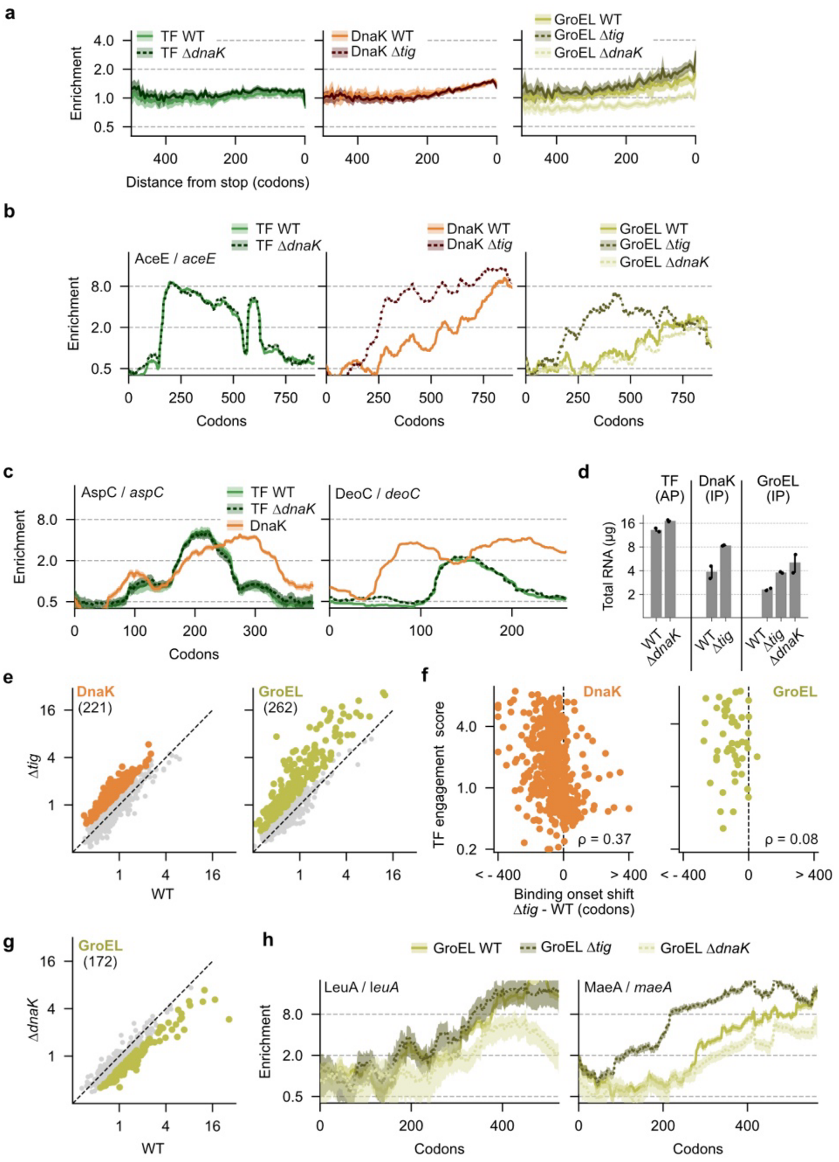

Supplementary Fig. 5 | Chaperone binding network. a, Genome-wide (n = 3961) metagene enrichment profiles for chaperones in different strain backgrounds identical to Fig. 5b. Profiles are aligned to the stop codon. Solid lines and shadows indicate means and 95 % CI, respectively. **b**, Chaperone enrichment profiles to nascent AceE in different strain backgrounds. Solid lines indicate averages, shadows indicate 95 % CI. **c**, Chaperone enrichment profiles to nascent AspC and DeoC in different strain backgrounds. Solid lines indicate averages, shadows indicate 95 % CI. **d**, Amount of co-purified ribosomes upon TF affinity purification (AP), and DnaK or GroEL immunoprecipitation (IP), determined by RNA quantification (Bioanalyzer). Mean (bar height) and SD (error bars) are shown for two replicates (dots). **e**, Scatter plots compare total chaperone enrichments (RPM IP / RPM total) in WT (x-axis) and *Δtig* (y-axis) cells. Stronger DnaK (left, n = 644) and GroEL (right, n = 653) enrichment upon TF deletion (> 1.41-fold increase) is detected for 221 and 262 high affinity TF substrates, respectively. **f**, Altered engagement onsets upon TF deletion (x-axis) is plotted against TF engagement scores (y-axis) for all high affinity substrates of DnaK (left, n = 544) and GroEL (right, n = 50). ρ indicates the Spearman correlation coefficient. **g**, Scatter plot compares total chaperone enrichments in WT (x-axis) and *ΔdnaK* (y-axis) cells (n = 365). Weaker GroEL

enrichment upon DnaK deletion (< 1.41-fold decrease) is detect for 172 high affinity DnaK substrates. **h**, GroEL enrichment profiles to nascent LeuA and MaeA in different strain backgrounds. Solid lines indicate averages, shadows indicate 95 % CI. Source data are provided as a Source Data file.

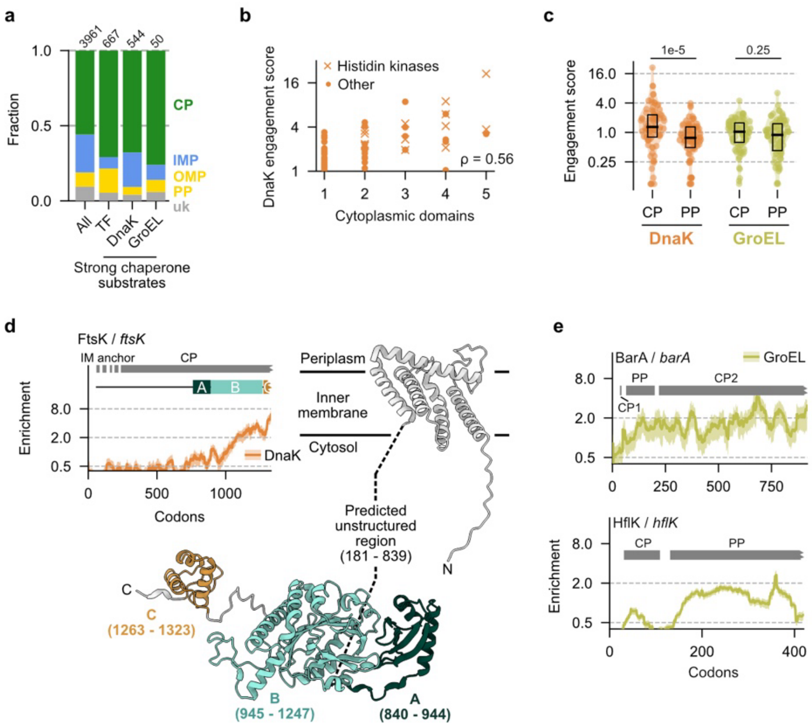

Supplementary Fig. 6 | Chaperone binding to nascent inner membrane proteins. a, Fractions of cellular localizations (cytoplasm, CP: green; inner membrane, IMP: blue; outer membrane, OMP: yellow; periplasm, PP: yellow; unknown, uk: grey) for all proteins (left) and strong chaperone substrates (n is indicated above bars). **b**, Scatterplot comparing the number of domains within cytoplasmic segments (x-axis) with the maximal DnaK engagement scores within these segments (y-axis) for strongly bound IMPs (n = 72). ρ indicates the spearman correlation coefficient. **c**, Highest DnaK (left) and GroEL (right) engagement scores within cytoplasmic (CP, n = 83) or periplasmic (PP, n = 85) segments of IMPs larger than 150 amino acids. Black bars indicate medians and boxes indicate the interquartile range. P-values were calculated using two-sided Mann–Whitney U tests. **d**, Same as Fig. 5g but for FtsK (AF-P46889). IM anchor = inner membrane anchor with four α-helices. Amino acids from 181 to 839 are predicted to be unstructured and are not shown. Colors indicate the domain annotation of the cytosolic C-terminus. **e,** GroEL enrichment profiles for nascent BarA (top) and HflK (bottom). Grey bars indicate membrane topology of loop segments (CP: cytoplasmic; PP: periplasmic). Solid lines indicate averages, shadows indicate 95 % CI. Source data are provided as a Source Data file.

Supplementary Figure 1a

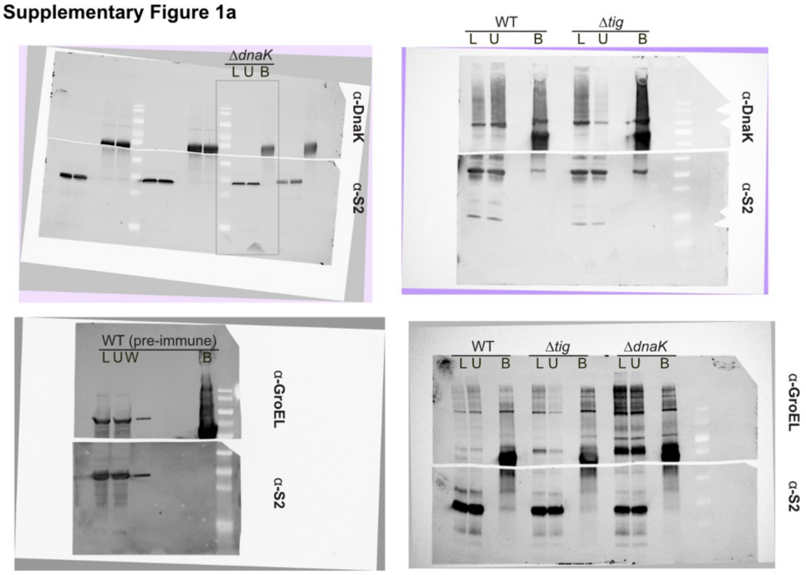

Supplementary Figure 2e

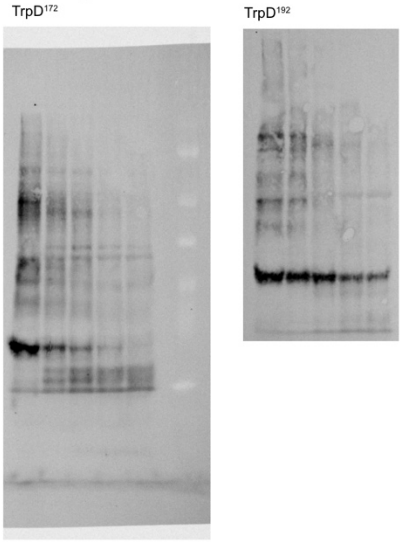

Supplementary Fig. 7 | Raw western blot files

Fig. 2b: AF-P36548_v3

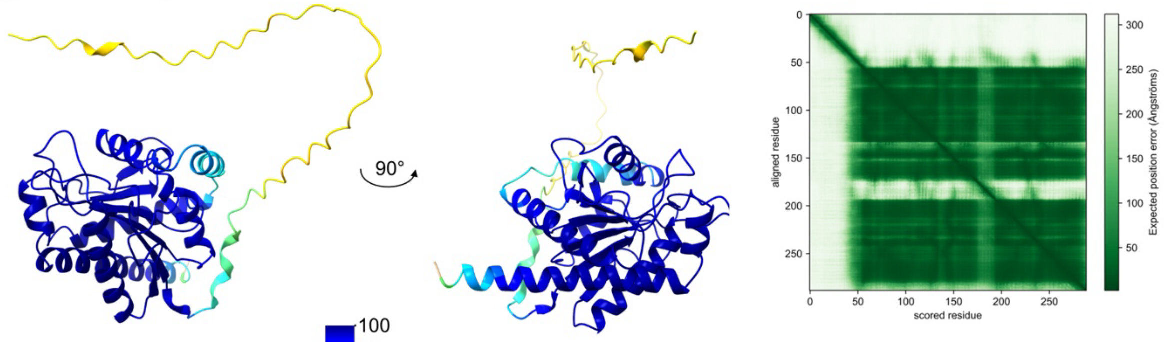

Fig. 2d: AF-P0AES4_v3

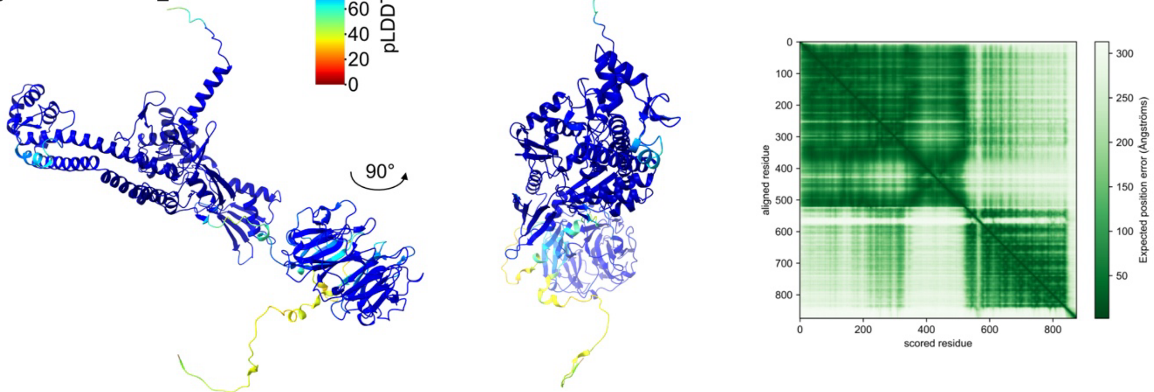

Fig. 4c: AF-P37744_v3

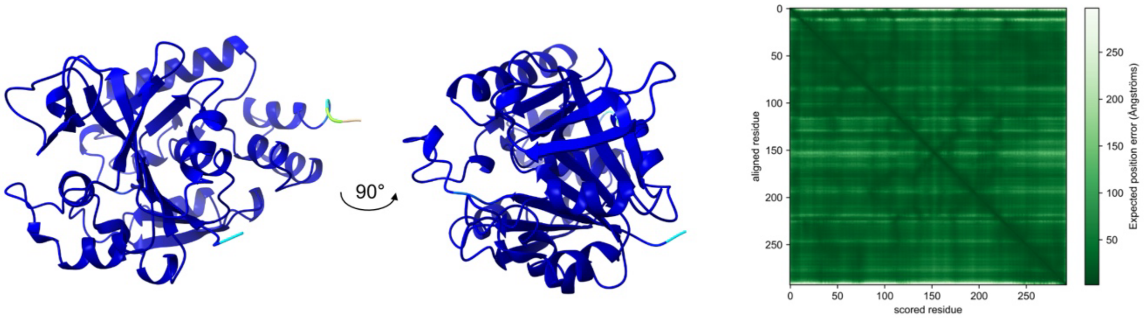

Fig. 4e: AF-P0AFG8_v3

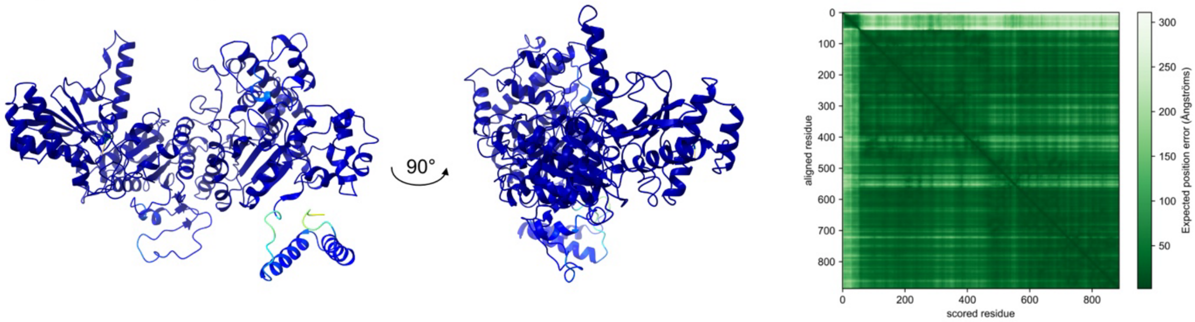

Fig. 6b: AF-P0AEC5_v3

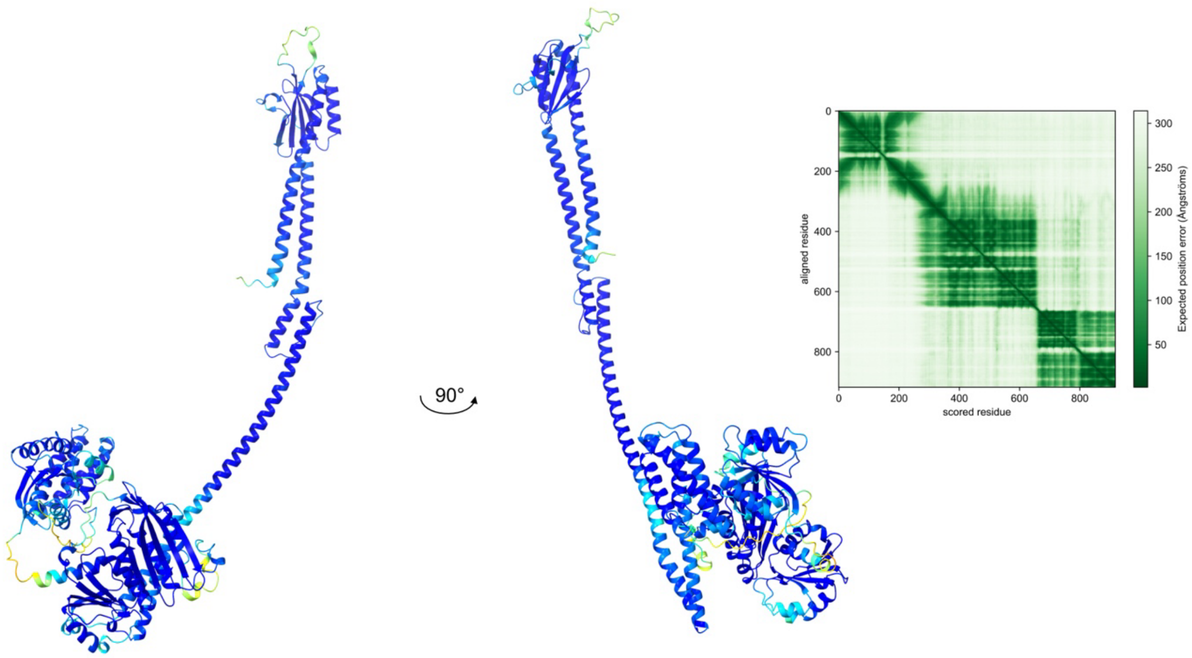

Fig. S2e: AF-P00904_v3

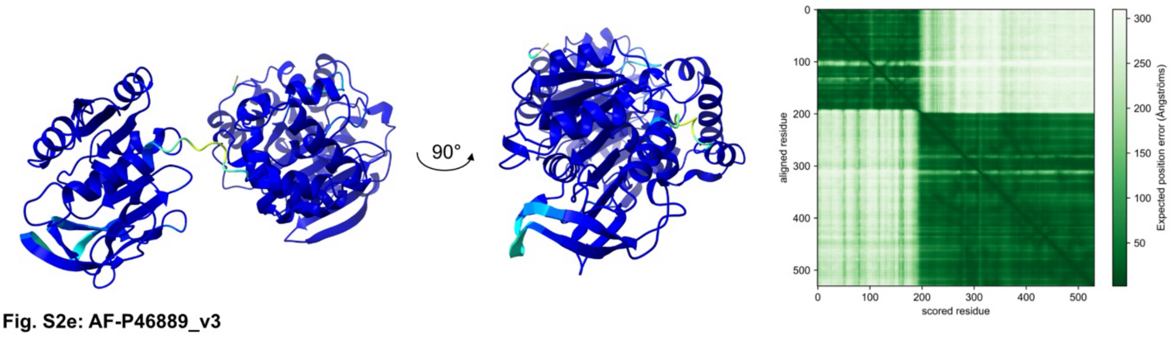

Fig. S2e: AF-P46889_v3

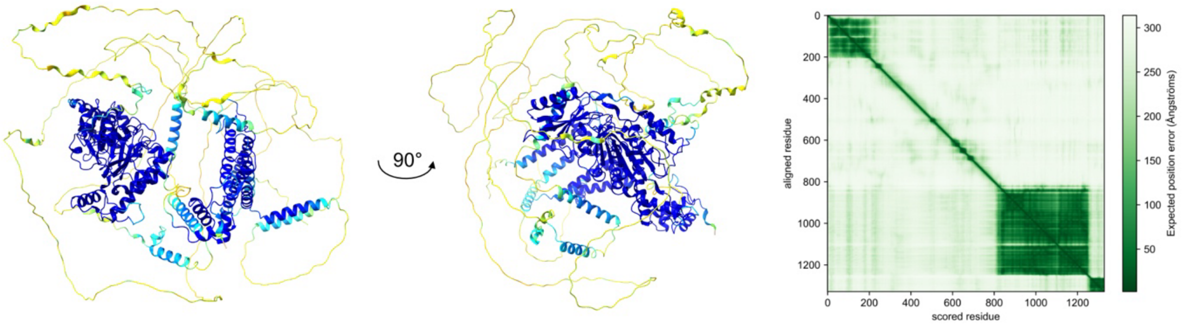

Supplementary Fig. 8 | AlphaFold Model data

Supplementary Table 1: Used primer and reagents

**REAGENT or RESOURCE**	**SOURCE**	**IDENTIFIER**
Antibodies		
Rabbit polyclonal against the ATPase domain of DnaK	Mogk *et al*. 1999	N/A
Rabbit polyclonal anti-GroEL	Laboratory Collection	N/A
Sheep polyclonal anti-RpS2	Laboratory Collection	N/A
**Bacterial and Virus Strains**		
E. coli MC4100	Laboratory Collection	N/A
E. coli MC4100 Δ*tig*::*kan*	Laboratory Collection	N/A
Escherichia coli MC4100 Δ*dnak*::*cam*	Laboratory Collection	N/A
Escherichia coli MC4100 Δ*tig*::*tig*-TEV-AviTag	Laboratory collection	N/A
Escherichia coli MC4100 Δ*dnak* Δ*tig*::*tig*-TEV-AviTag	This study	N/A
**Chemicals, Peptides, and Recombinant Proteins**		
Hexokinase	Sigma-Aldrich	Cat. no. 9001-51-8
Strep-Tactin Sepharose	IBA, Germany	Cat. no. 2-1201-025

Dynabeads Protein A	Thermo Fisher Scientific	Cat. no. 10002D
Streptavidin magnetic beads	New England BioLabs	Cat. no. S1420S
CircLigase ssDNA ligase	Epicentre	Cat. no. 76081-610
Micrococcal nuclease (S7 nuclease)	Laboratory Collection (Becker, et al.)	N/A
Opti-Taq DNA polymerase	EURx Ltd	Cat. no. E2600-03
Phusion_High-Fidelity DNA polymerase	New England BioLabs	Cat. no. M0530L
RNase-free Dnase I	Roche	Cat. no. 03539121103
Superscript III Reverse Transcriptase	Invitrogen	cat.no. 18080093
T4 DNA polynucleotide kinase	New England BioLabs	Cat. no. M0201L
T4 RNA ligase 2, truncated	New England BioLabs	Cat. no. M0242L
10 bp DNA Ladder	Invitrogen	Cat. no. 10821015
cOmpleteTM, EDTA-free Protease Inhibitor Cocktail	Sigma-Aldrich	Cat. no. 11873580001
**Critical Commercial Assays**		
Agilent High Sensitivity DNA kit	Agilent Technologies	Cat. no. 5067-4626
Agilent RNA 600 NANO kit	Agilent Technologies	Cat. no. 5067-1511
Agilent Small RNA kit	Agilent Technologies	Cat. no. 5067-1548
QubitTM dsDNA HS Assay	Thermo Fisher Scientific	Cat. no. Q32854
**Oligonucleotides**		
Linker L1<br>5'_App/CTGTAGGCACCATCAAT/3ddC_3'	IDT (Becker et al. 2013)	N/A
Linker L1'L2'<br>5'_5phos/GATCGTCGGACTGTAGAACTCTGAACCTGTCG GTGGTCGCCGTATCATT/ iSp18/CACTCA/iSp18/CAAGCAGAAGACGGCATACGAAT TGATGGTGCCTACAG_3'	IDT (Becker et al. 2013)	N/A
PCR primer 1 (reverse)<br>5'_CAAGCAGAAGACGGCATACGA_3'	IDT (Becker et al. 2013)	N/A

Barcoding primer Forward index1<br>5’_AATGATACGGCGACCACCGAGATCGGAAGAGCACAC<br>GTCTGAACTCCAGTCACATCACGCGACAGGTTCAGAGTT<br>C_3’	IDT (Becker et al. 2013)	N/A
Barcoding primer Forward index2<br>5’_AATGATACGGCGACCACCGAGATCGGAAGAGCACAC<br>GTCTGAACTCCAGTCACTGACCACGACAGGTTCAGAGTT<br>C_3’	IDT (Becker et al. 2013)	N/A
Barcoding primer Forward index3<br>5’_AATGATACGGCGACCACCGAGATCGGAAGAGCACAC<br>GTCTGAACTCCAGTCACCAGATCCGACAGGTTCAGAGTT<br>C_3’	IDT (Becker et al. 2013)	N/A
Barcoding primer Forward index4<br>5’_AATGATACGGCGACCACCGAGATCGGAAGAGCACAC<br>GTCTGAACTCCAGTCACGCCAATCGACAGGTTCAGAGTT<br>C_3’	IDT (Becker et al. 2013)	N/A
Barcoding primer Forward index5<br>5’_AATGATACGGCGACCACCGAGATCGGAAGAGCACAC<br>GTCTGAACTCCAGTCACACTTGACGACAGGTTCAGAGTT<br>C_3’	IDT (Becker et al. 2013)	N/A
Barcoding primer Forward index6<br>5’_AATGATACGGCGACCACCGAGATCGGAAGAGCACAC<br>GTCTGAACTCCAGTCACGATCAGCGACAGGTTCAGAGTT<br>C_3’	IDT (Becker et al. 2013)	N/A
Barcoding primer Forward index7<br>5’_AATGATACGGCGACCACCGAGATCGGAAGAGCACAC<br>GTCTGAACTCCAGTCACGGCTACCGACAGGTTCAGAGTT<br>C_3’	IDT (Becker et al. 2013)	N/A
Barcoding primer Forward index8<br>5’_AATGATACGGCGACCACCGAGATCGGAAGAGCACAC<br>GTCTGAACTCCAGTCACCTTGTACGACAGGTTCAGAGTTC<br>_3’	IDT (Becker et al. 2013)	N/A
Barcoding primer Forward index9<br>5’_AATGATACGGCGACCACCGAGATCGGAAGAGCACAC<br>GTCTGAACTCCAGTCAC AGATCG<br>CGACAGGTTCAGAGTTC_3’	IDT (Becker et al. 2013)	N/A

Barcoding primer Forward index10<br>5’_AATGATACGGCGACCACCGAGATCGGAAGAGCACAC<br>GTCTGAACTCCAGTCAC GCCTAT<br>CGACAGGTTCAGAGTTC_3’	IDT (Becker et al. 2013)	N/A
Barcoding primer Forward index11<br>5’_AATGATACGGCGACCACCGAGATCGGAAGAGCACAC<br>GTCTGAACTCCAGTCAC TGGTCA<br>CGACAGGTTCAGAGTTC_3’	IDT (Becker et al. 2013)	N/A
Barcoding primer Forward index12<br>5’_AATGATACGGCGACCACCGAGATCGGAAGAGCACAC<br>GTCTGAACTCCAGTCAC CACTGT<br>CGACAGGTTCAGAGTTC_3’	IDT (Becker et al. 2013)	N/A
Barcoding primer Forward index13<br>5’_AATGATACGGCGACCACCGAGATCGGAAGAGCACAC<br>GTCTGAACTCCAGTCAC ATTGGC<br>CGACAGGTTCAGAGTTC_3’	IDT (Becker et al. 2013)	N/A
Barcoding primer Forward index14<br>5’_AATGATACGGCGACCACCGAGATCGGAAGAGCACAC<br>GTCTGAACTCCAGTCAC GCTCTG<br>CGACAGGTTCAGAGTTC_3’	IDT (Becker et al. 2013)	N/A
Barcoding primer Forward index15<br>5’_AATGATACGGCGACCACCGAGATCGGAAGAGCACAC<br>GTCTGAACTCCAGTCAC GGACGG<br>CGACAGGTTCAGAGTTC_3’	IDT (Becker et al. 2013)	N/A
Barcoding primer Forward index16<br>5’_AATGATACGGCGACCACCGAGATCGGAAGAGCACAC<br>GTCTGAACTCCAGTCAC AAGCTA<br>CGACAGGTTCAGAGTTC_3’	IDT (Becker et al. 2013)	N/A

Supplementary References

1. Ward JH. Hierarchical Grouping to Optimize an Objective Function. *Journal of the American Statistical Association* **58**, (1963).

2. Cock PJ, *et al.* Biopython: freely available Python tools for computational molecular biology and bioinformatics. *Bioinformatics* **25**, 1422-1423 (2009).

3. Kyte J, Doolittle RF. A simple method for displaying the hydropathic character of a protein. *J Mol Biol* **157**, 105-132 (1982).
