# Supplementary material for: Proteome-wide determinants of co-translational chaperone binding in bacteria
Source: Nat Commun. 2025 May 10;16:4361. doi: 10.1038/s41467-025-59067-9 (PMC12065913; doi:10.1038/s41467-025-59067-9)
Supplement: Supplementary file 2 — Description of Additional Supplementary Files [file 41467_2025_59067_MOESM2_ESM.pdf]

Description of Additional Supplementary Files

File Name: Supplementary Data 1

Description: Ranked lists of TF, DnaK and GroEL nascent chain interactors.

File Name: Supplementary Data 2

Description: Strong chaperone substrates included in clusters A.

File Name: Supplementary Data 3

Description: Significantly enriched CATH-domains (top) and GO-Terms (bottom) among 134 cluster A genes of TF, DnaK and GroEL.
