# Supplementary material for: Proteome-wide determinants of co-translational chaperone binding in bacteria
Source: Nat Commun. 2025 May 10;16:4361. doi: 10.1038/s41467-025-59067-9 (PMC12065913; doi:10.1038/s41467-025-59067-9)
Supplement: Supplementary file 6 — Reporting Summary [file 41467_2025_59067_MOESM6_ESM.pdf]

nature portfolio

Corresponding author(s): Bernd Bukau
Günter Kramer
Sander Tans

Last updated by author(s): Mar 17, 2025

Reporting Summary

Nature Portfolio wishes to improve the reproducibility of the work that we publish. This form provides structure for consistency and transparency in reporting. For further information on Nature Portfolio policies, see our Editorial Policies and the Editorial Policy Checklist.

Statistics

For all statistical analyses, confirm that the following items are present in the figure legend, table legend, main text, or Methods section.

n/a	Confirmed	
☐	☒	The exact sample size (*n*) for each experimental group/condition, given as a discrete number and unit of measurement
☒	☐	A statement on whether measurements were taken from distinct samples or whether the same sample was measured repeatedly
☐	☒	The statistical test(s) used AND whether they are one- or two-sided<br>*Only common tests should be described solely by name; describe more complex techniques in the Methods section.*
☒	☐	A description of all covariates tested
☒	☐	A description of any assumptions or corrections, such as tests of normality and adjustment for multiple comparisons
☐	☒	A full description of the statistical parameters including central tendency (e.g. means) or other basic estimates (e.g. regression coefficient) AND variation (e.g. standard deviation) or associated estimates of uncertainty (e.g. confidence intervals)
☐	☒	For null hypothesis testing, the test statistic (e.g. *F*, *t*, *r*) with confidence intervals, effect sizes, degrees of freedom and *P* value noted<br>*Give P values as exact values whenever suitable.*
☒	☐	For Bayesian analysis, information on the choice of priors and Markov chain Monte Carlo settings
☒	☐	For hierarchical and complex designs, identification of the appropriate level for tests and full reporting of outcomes
☒	☐	Estimates of effect sizes (e.g. Cohen's *d*, Pearson's *r*), indicating how they were calculated

Our web collection on statistics for biologists contains articles on many of the points above.

Software and code

Policy information about availability of computer code

Data collection	HISeqTM 2000
Data analysis	Cutadapt 1.14<br>Bowtie1 version 1.3.1., Langmead and Salzberg, 2012<br>Python version 3.10 Python Software Foundation<br>numpy version 2.1.3<br>scipy version 1.15.1<br>tslearn version 0.6.3<br>statsmodels version 0.14.4<br>matplotlib version 3.10

For manuscripts utilizing custom algorithms or software that are central to the research but not yet described in published literature, software must be made available to editors and reviewers. We strongly encourage code deposition in a community repository (e.g. GitHub). See the Nature Portfolio guidelines for submitting code & software for further information.

Data

Policy information about availability of data

All manuscripts must include a data availability statement. This statement should provide the following information, where applicable:

- Accession codes, unique identifiers, or web links for publicly available datasets
- A description of any restrictions on data availability
- For clinical datasets or third party data, please ensure that the statement adheres to our policy

All data necessary to interpret, verify and extend the research (processed ribosome profiling data and chaperone enrichment confidence intervals) in this article have been deposited at the open science foundation (OSF) [https://doi.org/10.17605/OSF.IO/SH4YQ]. Raw sequencing data are accessible at the Gene Expression Omnibus (GEO) repository with the accession code GSE292386 [https://www.ncbi.xyz/geo/query/acc.cgi?acc=GSE292386]. Data is publicly available as of the date of publication. Unless otherwise stated, all data supporting the results of this study can be found in the article, supplementary, source data files or the OSF website. Source data are provided with this study.

Research involving human participants, their data, or biological material

Policy information about studies with human participants or human data. See also policy information about sex, gender (identity/presentation), and sexual orientation and race, ethnicity and racism.

Reporting on sex and gender	Does not apply
Reporting on race, ethnicity, or other socially relevant groupings	Does not apply
Population characteristics	Does not apply
Recruitment	Does not apply
Ethics oversight	Does not apply

Note that full information on the approval of the study protocol must also be provided in the manuscript.

Field-specific reporting

Please select the one below that is the best fit for your research. If you are not sure, read the appropriate sections before making your selection.

- [x] Life sciences
- [ ] Behavioural & social sciences
- [ ] Ecological, evolutionary & environmental sciences

For a reference copy of the document with all sections, see nature.com/documents/nr-reporting-summary-flat.pdf

Life sciences study design

All studies must disclose on these points even when the disclosure is negative.

Sample size	Does not apply
Data exclusions	At the read processing step of demultiplexed deep sequencing datasets low-quality reads, reads that are either too short or too long, as well as reads of non-coding RNAs, including rRNAs and snoRNAs were removed. At the genome read alignment step, reads that present more than two mismatches where exclude. These exclusion criteria were pre-established by multiple previous publication from our and other labs.
Replication	two biological replicates
Randomization	Does not apply
Blinding	Does not apply

Reporting for specific materials, systems and methods

We require information from authors about some types of materials, experimental systems and methods used in many studies. Here, indicate whether each material, system or method listed is relevant to your study. If you are not sure if a list item applies to your research, read the appropriate section before selecting a response.

Materials & experimental systems

n/a	Involved in the study
☐	☒ Antibodies
☒	☐ Eukaryotic cell lines
☒	☐ Palaeontology and archaeology
☒	☐ Animals and other organisms
☒	☐ Clinical data
☒	☐ Dual use research of concern
☒	☐ Plants

Methods

n/a	Involved in the study
☒	☐ ChIP-seq
☒	☐ Flow cytometry
☒	☐ MRI-based neuroimaging

Antibodies

Antibodies used	Rabbit polyclonal against the ATPase domain of DnaK, Mogk et al. 1999 . Rabbit polyclonal anti-GroEL Laboratory Collection. Sheep polyclonal anti-RpS2 Laboratory Collection.
Validation	Antibodies were validated by previous lab members and were already used in multiple studies of the Bukaulab

Plants

Seed stocks	Does not apply
Novel plant genotypes	Does not apply
Authentication	Does not apply
